# ADVANCING DIVERSITY IN ADRD/AGING RESEARCH AND CLINICAL CARE: EDUCATIONAL AND CAREER TRAJECTORIES OF MSTEM MENTEES

**DOI:** 10.1093/geroni/igac059.3068

**Published:** 2022-12-20

**Authors:** Sheri Thompson

**Affiliations:** UC San Diego, La Jolla, California, United States

## Abstract

As an NIA-funded mentorship program, MADURA addresses the lack of diversity among Aging and Alzheimer’s Disease & Related Dementia (ADRD) researchers and clinicians. Aims include improving retention and academic success of URM MSTEM undergraduates, and increasing rates of graduate/medical school applications and entry into Aging/ADRD clinical careers. MADURA offers paid research lab experience, weekly whole-cohort research skills training, guest seminars and presentations, and weekly faculty-facilitated small group supervision, advising and support. It served 23–29 undergraduates/quarter, in its initial five quarters. The Program accepts students at varying undergraduate educational levels, particularly because students from target populations often enter as Junior-level transfers from community colleges. Thus, although the Program just completed its second year, 17 MADURA trainees have already graduated. While ongoing data collection on Program, Mentor and Student Mentee performance is beneficial, outcomes data are of utmost importance to ensuring achievement of aims. This poster will present descriptive data on MADURA graduates’ immediate employment and educational activities. Notable findings include a significant proportion of trainees who take a gap year before applying to graduate or medical school (for financial and personal reasons), and others taking internships, post-baccalaureate training or research jobs, to strengthen future medical and graduate school applications. Understanding the multiple pathways of recent graduates will enable mentorship programs to help current trainees critique and optimize preparations for their selected educational and career trajectories. These findings also suggest additional mentorship program outcomes of interest (beyond a sole focus on graduate program acceptance), and the need for longer-term alumni follow-up.

